# *In Vitro* Activity of Ertapenem against Neisseria gonorrhoeae Clinical Isolates with Decreased Susceptibility or Resistance to Extended-Spectrum Cephalosporins in Nanjing, China (2013 to 2019)

**DOI:** 10.1128/aac.00109-22

**Published:** 2022-05-02

**Authors:** Xuechun Li, Wenjing Le, Xiangdi Lou, Caroline A. Genco, Peter A. Rice, Xiaohong Su

**Affiliations:** a Institute of Dermatology, Chinese Academy of Medical Sciences and Peking Union Medical College, Nanjing, Jiangsu, China; b Department of Immunology, School of Medicine, Tufts University, Boston, Massachusetts, USA; c Division of Infectious Diseases and Immunology, Department of Medicine, University of Massachusetts Chan Medical School, Worcester, Massachusetts, USA

**Keywords:** *Neisseria gonorrhoeae*, ertapenem, ESCs, resistance

## Abstract

Neisseria gonorrhoeae isolates collected in Nanjing, China, that possessed decreased susceptibility (or resistance) to extended-spectrum cephalosporins (ESCs) were examined for susceptibility to ertapenem, and their sequence types were determined. Ceftriaxone and cefixime MICs of ≥0.125 mg/L and ≥0.25 mg/L, respectively, were first determined in 259 strains isolated between 2013 and 2019, and then MICs of ertapenem were measured using the antimicrobial gradient Epsilometer test (Etest). Also, genetic determinants of ESC resistance were identified and N. gonorrhoeae multiantigen sequence typing (NG-MAST) was performed to analyze associations with ertapenem susceptibility. All isolates displayed ertapenem MICs between 0.006 mg/L and 0.38 mg/L; the overall MIC_50_ and MIC_90_ were 0.032 mg/L and 0.125 mg/L, respectively. Forty-four (17.0%) isolates displayed ertapenem MICs of ≥0.125 mg/L; 10 (3.9%) had MICs of ≥0.25 mg/L. The proportion of isolates with ertapenem MICs of ≥0.125 mg/L increased from 4.0% in 2013 to 20.0% in 2019 (χ^2^ = 24.144, *P* < 0.001; chi-square test for linear trend). The *penA* mosaic allele was present in a significantly higher proportion of isolates with ertapenem MICs of ≥0.125 mg/L than of isolates with MICs of ≤0.094 mg/L) (97.7% versus 34.9%, respectively; χ^2^ = 58.158, *P* < 0.001). ST5308 was the most prevalent NG-MAST type (8.5%); ST5308 was also significantly more common among isolates with ertapenem MICs of ≥0.125 mg/L than isolates with MICs of ≤0.094 mg/L (22.7% and 5.6%, respectively; χ^2^ = 13.815, *P* = 0.001). Ertapenem may be effective therapy for gonococcal isolates with decreased susceptibility or resistance to ESCs and isolates with identifiable genetic resistance determinants.

## INTRODUCTION

Gonorrhea is the second most common bacterial sexually transmitted infection (STI) and a major global public health problem. The World Health Organization (WHO) estimated that 87 million new cases occurred worldwide in adults aged 15 to 49, in 2016 (1). In China, the reported incidence of gonorrhea increased by 36.03% (7.05 to 9.59 cases per 100,000 population) from 2014 to 2018 (2). Treatment of gonorrhea is challenging because Neisseria gonorrhoeae has developed resistance to most antimicrobials (AMR) that have been used for therapy, including sulfonamides, penicillins, tetracyclines, fluoroquinolones, and early-generation and, rarely, extended-spectrum cephalosporins (ESCs) (3–7).

Currently, ceftriaxone monotherapy or dual therapy with ceftriaxone or cefixime plus azithromycin has been recommended as first-line treatment of uncomplicated gonorrhea in most countries (8–10). In the United States (U.S.), azithromycin is no longer recommended as part of a first-line regimen (10) because of increased incidence of azithromycin resistance (from 0.6% in 2013 to 4.6% in 2018) (11). The U.S. Centers for Disease Control and Prevention (CDC)-recommended dose of ceftriaxone was doubled in 2020 (from 250 mg to 500 mg intramuscularly [i.m.]) (10) based on doubling of MICs of current strains compared with MICs 20 years ago (11). Modeling of urogenital concentrations of ceftriaxone that estimate the 250-mg dose does not predict a concentration for 24 h that is higher than the MIC of ceftriaxone for most current U.S. strains of N. gonorrhoeae (12). Ceftriaxone concentrations in the pharynx are variable, and treatment of N. gonorrhoeae may require longer times to achieve necessary MICs in the pharynx (13, 14). In the United Kingdom, the emergence of azithromycin resistance (9.2% in 2017), the increase in the modal ceftriaxone MIC distribution (15), and the identification of isolates resistant to both ceftriaxone and azithromycin (16, 17) prompted a revision of recommendations in 2018 from dual therapy with ceftriaxone and azithromycin to therapy with a higher dose of ceftriaxone (1 g) alone (18). Based on the emergence of gonococcal isolates that displayed decreased susceptibility to ceftriaxone in China (10.8% in 2013 to 2016) (19) and the identification of the ceftriaxone-resistant N. gonorrhoeae strain FC428 (20), now present worldwide, the dose of ceftriaxone recommended by the China CDC was also increased in 2020, from 250 mg to 1 g (21).

Ertapenem is a parenteral carbapenem, effective against Gram-negative bacteria that may, otherwise, be resistant to cephalosporins. Similar to other β-lactams, ertapenem inhibits cell wall synthesis by binding to and inhibiting penicillin-binding proteins (PBPs) (22). It is well tolerated and effective and has a safety profile comparable to that of ceftriaxone (23, 24). Ertapenem has been used successfully to treat N. gonorrhoeae with both high-level azithromycin and high-level ceftriaxone resistance (17). A recently reported randomized treatment trial showed that in a primary per-protocol analysis, a single 1-g dose of ertapenem was 99% effective (one treatment failure) for treatment of uncomplicated anogenital gonorrhea, noninferior to a single 500-mg dose of ceftriaxone (100% effective). All N. gonorrhoeae strains were susceptible to ceftriaxone (MICs of ≤0.012 mg/L) and also displayed low MICs for ertapenem (MICs of ≤0.008 mg/L) (25).

No specific genetic determinants of ertapenem resistance or carbapenemases, generally, have been identified in N. gonorrhoeae; however, there may be overlap with resistance mechanisms exhibited toward other ESCs (26). Mechanisms of resistance against ESCs can result from amino acid changes caused by nucleotide mutations in *penA* (encoding penicillin-binding protein 2 [PBP2]), *mtrR* (encoding the multiple transfer resistance repressor [MtrR]), *penB* (encoding porin PorB), and *ponA* (encoding penicillin-binding protein 1 [PBP1]) in N. gonorrhoeae (3, 27–30).

The major aim of the present study was to examine *in vitro* activity of ertapenem against N. gonorrhoeae isolates with decreased susceptibility (or resistance) to ESCs. We also identified ESC resistance determinants and their association with susceptibility of N. gonorrhoeae strains to ertapenem. N. gonorrhoeae multiantigen sequence typing (NG-MAST) of N. gonorrhoeae isolates was performed to assess distribution according to ertapenem MICs and, potentially, to identify clonality of isolates with increased resistance.

## RESULTS

### Antimicrobial susceptibility.

A total of 259 N. gonorrhoeae isolates with decreased susceptibility or resistance to ceftriaxone and/or cefixime were identified. The MIC ranges of ceftriaxone and cefixime for these isolates were 0.06 to 1 mg/L (MIC_50_, 0.125 mg/L, and MIC_90_, 0.125 mg/L) and 0.06 to ≥4 mg/L (MIC_50_, 0.125 mg/L, and MIC_90_, 0.5 mg/L), respectively. Among these isolates, 9 (3.5%) were fully resistant to ceftriaxone (MICs of ≥0.5 mg/L) and cefixime (MICs of ≥2 mg/L).

**FIG 1:**
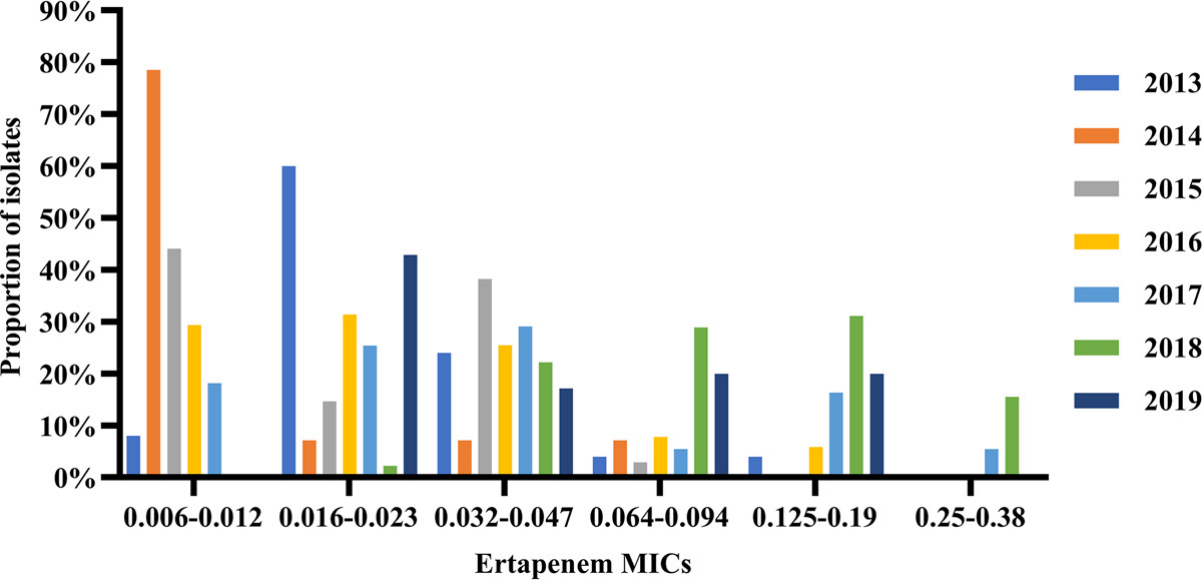
Distribution of ertapenem MICs (milligrams per liter) for 259 Neisseria gonorrhoeae clinical isolates with decreased susceptibility (or resistance) to ESCs isolated in Nanjing, China, 2013 to 2019.

MICs of ertapenem against the 259 isolates ranged from 0.006 mg/L to 0.38 mg/L; MIC_50_ and MIC_90_ were 0.032 mg/L and 0.125 mg/L, respectively. For the 9 N. gonorrhoeae isolates fully resistant to ceftriaxone (MICs of ≥0.5 mg/L) and cefixime (MICs of ≥2 mg/L), the ertapenem MIC_50_, MIC_90_, and MIC range were 0.094 mg/L, 0.19 mg/L, and 0.023 to 0.19 mg/L, respectively. Forty-four (17.0%) isolates had ertapenem MICs of ≥0.125 mg/L and 10 (3.9%) had MICs of ≥0.25 mg/L, MICs that represent the WHO-recommended susceptibility breakpoints for ceftriaxone and cefixime, respectively. The ertapenem MIC_50_ and MIC_90_ increased from 0.023 mg/L and 0.047 mg/L in 2013 to 0.047 mg/L and 0.125 mg/L in 2019, respectively. The distributions of ertapenem MICs during 2013 to 2019 are shown in Fig. 1. The proportion of isolates with ertapenem MICs of ≥0.125 mg/L (the breakpoint against ceftriaxone) increased from 4.0% in 2013 to 20.0% in 2019, showing an overall upward trend during the study period (χ^2^ = 24.144, *P* < 0.001; chi-square test for linear trend), while the percentage of isolates with MICs of ≤0.012 mg/L declined in each successive year, sequentially (χ^2^ = 23.634, *P* < 0.001; chi-square test for linear trend).

### Genetic resistance determinants (*penA*, *mtrR*, *penB*, and *ponA*) for ESCs.

A *penA* mosaic allele was present in 118 (45.6%) N. gonorrhoeae isolates with decreased susceptibility or resistance to ESCs; nonmosaic *penA* alleles with A501V/T mutations were present in 139 (53.7%); the remaining 2 isolates (0.8%) possessed a nonmosaic allele with an A517G mutation. Mutations in the promoter and/or coding regions of the *mtrR* gene were identified in 179 (69.1%) isolates. Amino acid substitutions at residue G120 of the *penB* gene were present in 5 (1.9%) isolates; G120/A121 double mutations were present in 253 (97.7%). An L421P mutation in the *ponA* gene was present in 256 (98.8%) isolates.

Ertapenem susceptibilities of isolates containing the *penA* mosaic allele were lower than susceptibilities of isolates that lacked the mosaic allele. The MIC_50_, MIC_90_, and MIC range of ertapenem in strains with the *penA* mosaic allele were 0.047 mg/L, 0.19 mg/L, and 0.008 to 0.38 mg/L, respectively. Strains that lacked the mosaic allele had a MIC_50_, MIC_90_, and MIC range of ertapenem of 0.016 mg/L, 0.064 mg/L, and 0.006 to 0.125 mg/L, respectively. The *penA* mosaic allele was more common among isolates with increased ertapenem MICs (≥0.125 mg/L) (WHO-recommended susceptibility breakpoint against ceftriaxone) than isolates with MICs of ≤0.094 mg/L (97.7% versus 34.9%, respectively; χ^2^ = 58.158, *P* < 0.001) (Table 1). All isolates with ertapenem MICs of ≥0.25 mg/L (WHO-recommended susceptibility breakpoint against cefixime) possessed the *penA* mosaic allele. Conversely, the proportion of isolates with ertapenem MICs of ≤0.094 mg/L that possessed A501V/T mutations, specifically, was higher than that of isolates with MICs of ≥0.125 mg/L (64.2% versus 2.3%, respectively; χ^2^ = 56.307, *P* < 0.001) (Table 1). The two isolates with A517G mutations had ertapenem MICs of ≤0.094 mg/L (Table 1). Additionally, the proportion of isolates that possessed the *penA* mosaic allele increased from 4.0% in 2013 to 68.6% in 2019, showing an upward trend during the study period (χ^2^ = 34.343, *P* < 0.001; chi-square test for linear trend).

*mtrR* mutations were present in 34.1% (15/44) of isolates with ertapenem MICs of ≥0.125 mg/L and in 76.3% (164/215) of isolates with ertapenem MICs of ≤0.094 mg/L (χ^2^ = 30.453, *P* < 0.001). A single A-deletion in the *mtrR* promoter was identified more often in isolates with ertapenem MICs of ≤0.094 mg/L than in isolates with MICs of ≥0.125 mg/L (χ^2^ = 9.090, *P* = 0.0026) (Table 1). There were no significant differences in the rates of A39T or G45D *mtrR* mutations in the coding region accompanied (or not) by an A-deletion in the promoter region. An exception was a G45D mutation accompanied by an A-deletion in the promoter, which accounted for 11.2% (24/215) of isolates with ertapenem MICs of ≤0.094 mg/L and no isolates with ertapenem MICs of ≥0.125 mg/L (χ^2^ = 5.413, *P* = 0.0186) (Table 1). All but two isolates with ertapenem MICs of ≥0.25 mg/L lacked the *mtrR* mutations; the two exceptions harbored a single A-deletion in the *mtrR* promoter or G45D mutation in the *mtrR* coding region.

**TABLE 1:** *penA* and *mtrR* mutations in isolates with MICs to ertapenem of either ≤0.094 mg/L or ≥0.125 mg/L

Resistance determinant	No. (%) of isolates with MIC:		χ^2^	*P* value^*a*^
	≤0.094 mg/L (*n* = 215)	≥0.125mg/L (*n* = 44)		
*penA*				
Mosaic allele	75 (34.9)	43 (97.7)	58.158	<0.001
A501V/T^*b*^	138 (64.2)	1 (2.3)	56.307	<0.001
A517G^*b*^	2 (0.9)	0	0.413	1
*mtrR*				
A-deletion in promoter region^*c*^	102 (47.4)	10 (22.7)	9.090	0.0026
A-deletion,^*c*^ A39T	3 (1.4)	3 (6.8)	4.747	0.0632
A-deletion,^*c*^ G45D	24 (11.2)	0 (0)	5.413	0.0186
A39T	8 (3.7)	1 (2.3)	0.228	1.0000
G45D	27 (12.6)	1 (2.3)	4.007	0.0585
WT^*d*^	51 (23.7)	29 (65.9)	30.453	<0.001

a *P* of <0.05 was considered significant in chi-square (χ^2^) or Fisher exact testing.

b Nonmosaic *penA* alleles.

c A (adenine) deletion in the 13-bp inverted-repeat sequence of the *mtrR* promoter.

d WT, wild type.

### NG-MAST.

The 259 N. gonorrhoeae isolates were assigned to 161 N. gonorrhoeae multiantigen sequence typing (NG-MAST) types, of which 68 have not been reported previously in the NG-MAST database. The most prevalent NG-MAST sequence type (ST) was ST5308 (*n* = 22; ertapenem MIC_50_, 0.094 mg/L), followed by ST7554 (*n* = 17; ertapenem MIC_50_, 0.032 mg/L), ST3356 (*n* = 7; ertapenem MIC_50_, 0.023 mg/L), ST270 (*n* = 7; ertapenem MIC_50_, 0.008 mg/L), and ST4539 (*n* = 7; ertapenem MIC_50_, 0.016 mg/L). Among all sequence types, ST5308 was predominant among isolates with MICs of ≥0.125 mg/L to ertapenem (10/44 [22.7%]); these isolates also showed the highest ertapenem MIC_50_ (0.094 mg/L). Furthermore, ST5308 was more common among isolates with MICs of ≥0.125 mg/L to ertapenem versus isolates with MICs of ≤0.094 mg/L (22.7% and 5.6%, respectively; χ^2^ = 13.815, *P* = 0.001). All ST5308 isolates had a *penA* mosaic allele, G120K plus A121D substitutions in *penB*, and L421P in *ponA* but no *mtrR* mutations.

## DISCUSSION

Neisseria gonorrhoeae is becoming increasingly resistant to currently used antimicrobial agents with the real prospect that untreatable gonorrhea may soon appear (9, 17). In the context of limited treatment options, alternative antimicrobials, new and repurposed, are needed urgently to ensure future successful treatments.

In our study, the MIC_50_ of ertapenem (0.032 mg/L) was substantially lower than those observed for both ceftriaxone and cefixime (0.125 mg/L). The MIC_90_ of ertapenem (0.125 mg/L) was similar to the MIC_90_ observed for ceftriaxone (0.125 mg/L) but lower than the cefixime MIC_90_ (0.5 mg/L). Unemo et al. (26) reported in 2012 that, generally, ertapenem and ceftriaxone MIC_50_s and MIC_90_s were similar: 0.032 mg/L (both) and 0.064 mg/L (ertapenem)/0.125 mg/L (ceftriaxone), respectively, in 257 N. gonorrhoeae clinical isolates with highly diverse ceftriaxone MIC values referred to WHO Collaborating Centers for STIs. Similarly, ertapenem MIC ranges were lower than ceftriaxone and cefixime MIC ranges in our study, also reported by Unemo et al. (26). In our study, 83.0% and 96.1% of isolates had ertapenem MICs below the ceftriaxone and cefixime breakpoints (0.125 mg/L and 0.25 mg/L), respectively, similar to the study by Xu et al. (31) that examined gonococcal isolates from eight provinces in China. In that study, 83.3% of 24 isolates with decreased susceptibility to ceftriaxone (MIC of ≥0.25 mg/L) exhibited ertapenem MIC values of <0.25 mg/L, the cefixime breakpoint. Unemo et al. (26) reported that all strains had ertapenem MICs of ≤0.125 mg/L, the ceftriaxone breakpoint. These results predict that ertapenem may be uniformly effective clinically in most instances because higher MICs are infrequent (our study and the study by Xu et al. [31]) or absent altogether (26). Further support for clinical efficacy is derived from activity of ertapenem against two extensively drug-resistant (XDR) N. gonorrhoeae strains, H041 and F89; both are highly resistant to cefixime (MIC range, 4 to 8 mg/L) and ceftriaxone (MIC range, 2 to 4 mg/L) (26). Ertapenem MICs were reported to be significantly lower (0.064 mg/L and 0.016 mg/L) for these two strains, respectively (26), corroborated in a separate study where F89 had an ertapenem MIC of 0.03 mg/L (32). In our study, ertapenem was also effective against the 9 N. gonorrhoeae isolates fully resistant to ceftriaxone (MICs of ≥0.5 mg/L) and cefixime (MICs of ≥2 mg/L). Nonetheless, several studies (26, 32, 33) have shown that ertapenem had no apparent *in vitro* advantage over ceftriaxone for N. gonorrhoeae isolates with lower ceftriaxone MICs.

Similar to other β-lactam antimicrobials, reduced activity of ertapenem against some bacteria is mediated by mutations in porin that result in aberrant function (34), upregulation of efflux pumps (35), and production of carbapenemases (36). However, resistance of N. gonorrhoeae to ertapenem is not fully defined. We used the ceftriaxone breakpoint (0.125 mg/L) to separate isolates with MICs of ≥0.125 mg/L to ertapenem from those with lower MICs (≤0.094 mg/L) to distinguish certain genetic characteristics of the *penA* allele and the *mtrR* promoter known to be present in many strains that possess decreased susceptibility (or resistance) to extended-spectrum cephalosporins (ESCs). We used 0.125 mg/L as a dividing point to better understand how ertapenem MICs might relate to gonococcal isolates known to be above/below the ceftriaxone breakpoint. Ultimately, however, determination of an ertapenem breakpoint will require treatment failures to occur in a clinical trial(s) coupled with MIC determinations of corresponding strains that fail ertapenem therapy. We found that *penB* and *ponA* resistance determinants were present across most strains, perhaps without a meaningful effect on ertapenem susceptibility. We also found that the presence of a *penA* mosaic allele was strongly associated with increased MICs of ertapenem, similar to findings reported by Unemo et al. (26). The proportion of isolates with increased ertapenem MICs (MICs of ≥0.125 mg/L) showed an increasing trend during the study period in the absence of clinical use, which may have been the result of the yearly increase in the proportion of isolates that contained the *penA* mosaic allele, also shown in our study.

Our study also showed that *mtrR* mutations were present in a higher percentage of isolates with ertapenem MICs of ≤0.094 mg/L than isolates with ertapenem MICs of ≥0.125 mg/L, different from another Chinese study, which showed that the *mtrR* promoter A-deletion was significantly associated with strains displaying an ertapenem MIC of >0.125 mg/L (37). Our study investigated the association between ertapenem susceptibility and known ESC resistance determinants; other antibiotic resistance determinants, such as the presence of mosaic sequences in the *mtr* locus, which is primarily related to azithromycin resistance (38–40), were not included in our study. A previous study showed that 14 gonococcal isolates with mosaic alleles in the *mtr* locus displayed resistance to azithromycin (MIC of >256 mg/L) but had low cephalosporin MICs (0.016 mg/L for both cefixime and ceftriaxone) (40). Furthermore, mutations in the promoter and/or coding regions of the *mtrR* gene (resulting in an overexpression of the MtrCDE efflux pump) were not associated with increased MICs of AMR N. gonorrhoeae to ertapenem in our study.

NG-MAST has been evaluated as a tool for predicting specific antimicrobial resistance phenotypes in N. gonorrhoeae isolates (41, 42). In our study, ST5308 was the most prevalent NG-MAST sequence type (ST) among the 259 isolates with decreased susceptibility or resistance to ESCs. In addition, ST5308 was the most highly represented ST in isolates with increased ertapenem MICs (≥0.125 mg/L). ST5308 isolates, possessing a *penA* mosaic allele, have been reported in Hong Kong and were associated with decreased susceptibility to oral ESCs (43). Between 2013 and 2017, ST5308 was the most common gonococcal type isolated in Guangdong, China (44).

In summary, *in vitro* susceptibility to ertapenem of Neisseria gonorrhoeae isolates with decreased susceptibility (or resistance) to ESCs suggests potential for future use of ertapenem as a treatment for antimicrobial-resistant infections. However, the *penA* mosaic allele, commonly associated with ESC resistance, was also associated with increased MICs of ertapenem. Continued surveillance of antimicrobial susceptibility of ertapenem supplemented by sequence typing and NG-MAST classification is warranted.

## MATERIALS AND METHODS

### Bacterial strains.

From January 2013 to December 2019, a total of 1,321 N. gonorrhoeae strains were isolated from men with symptomatic urethritis (urethral discharge and/or dysuria) attending the sexually transmitted disease (STD) clinic at the Institute of Dermatology, Chinese Academy of Medical Sciences, Nanjing, Jiangsu Province, China. Urethral exudates were collected with cotton swabs and immediately streaked onto modified Thayer-Martin (T-M) selective medium (Zhuhai DL Biotech Co. Ltd.) and incubated at 36°C in candle jars for 24 to 48 h. N. gonorrhoeae was identified by colonial morphology, Gram’s stain, and oxidase testing, which are sufficient to identify N. gonorrhoeae colonies isolated on selective medium, particularly for urethral samples from symptomatic men (45, 46). Gonococcal isolates were subcultured onto chocolate agar plates; pure colonies were swabbed, suspended in tryptone-based soy broth, and frozen (−80°C) until used for antimicrobial testing.

### Antimicrobial susceptibility testing.

Susceptibility testing for ceftriaxone and cefixime was performed by the agar dilution method according to Clinical and Laboratory Standards Institute (CLSI) guidelines (47). According to criteria for decreased susceptibility or resistance to ceftriaxone (MIC of ≥0.125 mg/L) and cefixime (MIC of ≥0.25 mg/L), defined by WHO (48), 259 strains were eligible for inclusion in this study. Ertapenem susceptibility among these isolates was determined by the Etest (Liofilchem, Italy) method, according to the manufacturer’s instructions (49). Strain WHO-P was used for quality control. No interpretative criteria have been provided by WHO and CLSI (or any other organization) for ertapenem susceptibility breakpoints against N. gonorrhoeae.

### Sequencing of resistance determinants (*penA*, *mtrR*, *penB*, and *ponA*) and N. gonorrhoeae multiantigen sequence typing (NG-MAST).

Genomic DNA was prepared from individual gonococcal isolates using the rapid bacterial genomic DNA isolation kit (DNA-EZ Reagents V All-DNA-Fast-Out; Sangon Biotech Co. Ltd., Shanghai, China). ESC resistance determinants *penA*, *mtrR*, *penB*, and *ponA* were amplified by PCR using published primers (50), and DNA sequencing was performed by Suzhou Genewiz Biotech Co. Ltd. The sequencing data were uploaded to the NG-STAR database (https://ngstar.canada.ca) to determine the ESC resistance determinants.

Genetic characterization was performed by N. gonorrhoeae multiantigen sequence typing (NG-MAST), which assigns sequence types (STs) based on a combination of two variable genes, *porB* and *tbpB* (51); allele numbers and sequence types (STs) were then assigned.

### Data analysis.

Chi-square (χ^2^) testing for linear trends was used to assess changes in ertapenem MICs during the study period. Chi-square or Fisher exact testing was used to determine the associations between ertapenem susceptibility and gonococcal genetic resistance determinants or N. gonorrhoeae multiantigen sequence types. SPSS version 26.0 was used for statistical analysis, and *P* values of <0.05 were considered significant.
